# Translation and adaptation of the German version of the Veterans Rand—36/12 Item Health Survey

**DOI:** 10.1186/s12955-021-01722-y

**Published:** 2021-05-04

**Authors:** Ines Buchholz, You-Shan Feng, Maresa Buchholz, Lewis E. Kazis, Thomas Kohlmann

**Affiliations:** 1 University Medicine Greifswald, Walther-Rathenau-Str. 48, 17475 Greifswald, Germany; 2 Department of Health Law, Policy and Management, School of Public Health, Boston University, 715 Albany St Talbot Building, Boston, MA 02118 USA; 3 Institute for Clinical Epidemiology and Applied Biometrics, Medical University of Tübingen, Tübingen, Germany

**Keywords:** Quality of life, Veterans Rand 36 Items Health Survey, SF-36, SF-12, Health assessment, Health-related quality of life

## Abstract

**Background:**

The translated and culturally adapted German version of the Veterans Rand 36 Items Health Survey (VR-36), and its short form, the VR-12 counterpart, were validated in a German sample of orthopedic (n = 399) and psychosomatic (n = 292) inpatient rehabilitation patients.

**Methods:**

The instruments were analyzed regarding their acceptance, distributional properties, validity, responsiveness and ability to discriminate between groups by age, sex and clinically specific groups. Eligible study participants completed the VR-36 (n = 169) and the VR-12 (n = 177). They also completed validated patient-reported outcome measures (PROs) including the Euroqol-5 Dimensions 5 Level (EQ-5D-5L); Depression, Anxiety and Stress Scale (DASS); Hannover Functional Abilities Questionnaire (HFAQ); and CDC Healthy Days. The VR-12 and the VR-36 were compared to the reference instruments MOS Short Form-12 Items Health Survey (SF-12) version 1.0 and MOS Short Form-36 Items Health Survey (SF-36) version 1.0, using percent of completed items, distributional properties, correlation patterns, distribution measures of known groups validity, and effect size measures.

**Results:**

Item non-response varied between 1.8%/1.1% (SF_VR-36_/RE_SF-36_) and 6.5%/8.6% (GH_VR-36_/GH_SF-36_). PCS was normally distributed (Kolmogorov–Smirnov tests: p > 0.05) with means, standard deviations and ranges very similar between SF-36 (37.5 ± 11.7 [13.8–66.1]) and VR-36 (38.5 ± 10.1 [11.7–67.8]), SF-12 (36.9 ± 10.9 [15.5–61.6]) and VR-12 (36.2 ± 11.5 [12.7–59.3]). MCS was not normally distributed with slightly differing means and ranges between the instruments (MCS_VR-36_: 36.2 ± 14.2 [12.9–66.6], MCS_SF-36_: 39.0 ± 15.6 [2.0–73.2], MCS_VR-12_: 37.2 ± 13.8 [8.4–70.2], MCS_SF-12_: 39.0 ± 12.3 [17.6–65.4]). Construct validity was established by comparing correlation patterns of the MCS_VR_ and PCS_VR_ with measures of physical and mental health. For both PCS_VR_ and MCS_VR_ there were moderate (≥ 0.3) to high (≥ 0.5) correlations with convergent (PCS_VR_: 0.55–0.76, MCS_VR_: 0.60–0.78) and small correlations (< 0.1) with divergent (PCS_VR_: < 0.12, MCS_VR_: < 0.16) self-report measures. Known-groups validity was demonstrated for both VR-12 and VR-36 (MCS and PCS) via comparisons of distribution parameters with significant higher mean PCS and MCS scores in both VR instruments found in younger patients with fewer sick days in the last year and a shorter duration of rehabilitation.

**Conclusions:**

The psychometric analysis confirmed that the German VR is a valid and reliable instrument for use in orthopedic and psychosomatic rehabilitation. Yet further research is needed to evaluate its usefulness in other populations.

**Supplementary Information:**

The online version contains supplementary material available at 10.1186/s12955-021-01722-y.

## Background

Health related quality of life (HRQoL) is a crucial outcome metric used in settings from clinical trials [1, 2] to population health surveillance [3–7]. The Veterans Rand questionnaire (VR) is a multi-attribute generic instrument measuring patient-reported HRQoL. The instrument has a long (VR-36) and a short form (VR-12), both measuring a physical component summary (PCS_VR_) and a mental component summary (MCS_VR_). The VR-36 also is comprised of eight scales, which correspond closely to the Medical Outcome Study (MOS) Short Form 36 version 1.0 (SF-36, [8–10]).

The VR instruments were created to address the veteran population in the United States (US) [11]. The Veterans Health Administration (VHA) is a national health care system, which serves over nine million military veterans in the US. It is one of the largest integrated health care systems in the US. This patient population has special medical needs, is older, poorer, sicker (with more diseases than veterans nationally) and has a higher percentage of men than the general adult population [12–14]. The creation of the VR instruments has been previously documented [13–16] and shown to be valid for the VA population [13, 17–27] as well as other general US populations [28–35]. The English-language VR instruments have become an integral part of registries [36] and studies of National U.S. health programs [18, 37, 38] including the evaluation of the Medicare Advantage Program by the Centers for Medicare and Medicaid Services (CMS). Advantages of the VR instruments include their validity in older and sicker populations, their availability (all instruments are in the public domain) and their strong psychometric properties across different and wide-ranging socio-demographic and clinical groups.

In this study, we translated and culturally adapted the VR-36 into the German language (Germany) and validated the VR-36 and VR-12 in a population of German patients undergoing inpatient rehabilitation. The German VR-36 and VR-12 were comprehensively validated and compared to the SF-36 and SF-12 in inpatient populations of orthopedic and psychosomatic rehabilitation patients (the two largest clinical indications of German inpatient rehabilitation patients).

The SF-36 and the SF-12 are considered gold standards of self-assessed generic health instruments and they have been extensively distributed and used across a wide range of countries, populations and purposes. They are recommended for measuring patient outcomes in the medical rehabilitation setting in Germany [39–42]. Since the field of medical rehabilitation has been one of the most common applications of the SF-36 in the German-speaking countries, it was important to compare the measurement properties of the VR instruments to the SF-instruments in this setting.

## Methods

The study was conducted in two phases: phase (A) translating and culturally adapting the original English VR-36 into the German language (Germany); and phase (B) validating the VR-36 and its short version, the VR-12, in a randomized prospective study of inpatient rehabilitation patients with orthopedic and psychosomatic conditions.

### Phase (A) translation and cultural adaptation of the German VR

**Fig. 1:**
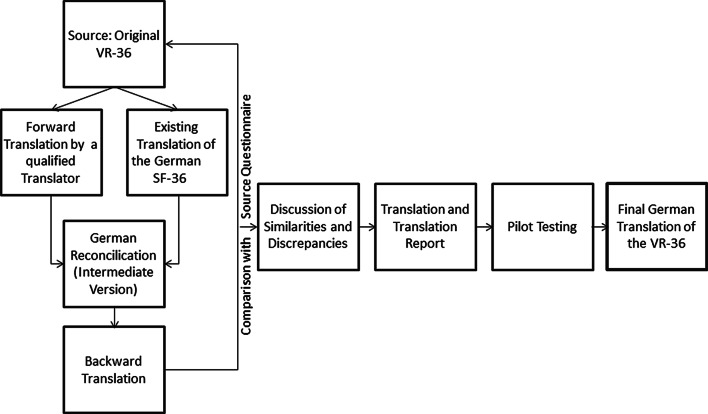
Flow chart of the translation process

The translation methodology followed a rigorous iterative forward–backward format to maintain the conceptual, functional, linguistic and cultural equivalence between the original (English) and the adapted (German) questionnaire. The translation procedure is summarized in Fig. 1. First, a German translation of the VR-36 was produced from the English original version by an experienced translator (DB). Because the VR-36 is analogous to the SF-36, the official German translation of the SF-36 items, which has already undergone rigorous translation and adaptation, served as a second translation to which we compared the forward translated VR items (German SF-36 Version 1 [8–10] and Version 2 [43]). A reconciled German VR-36 was produced after discussion of agreements and disagreements between the forward translation, SF-36 Version 1 and SF-36 Version 2, and translated back into the source language (English) by an experienced translator who is a native speaker of English and fluent in German. The backward translation was compared to the original English VR-36. Any discrepancies between the back translation and the English VR-36 were addressed with the back translator to determine the origins of discrepancies in the first reconciled German VR-36. After this stage, a pre-final version was produced, which was tested in a cognitive debriefing process with 26 patients and finalized afterwards.

### Phase (B) validation study

#### Patient recruitment

**Fig. 2:**
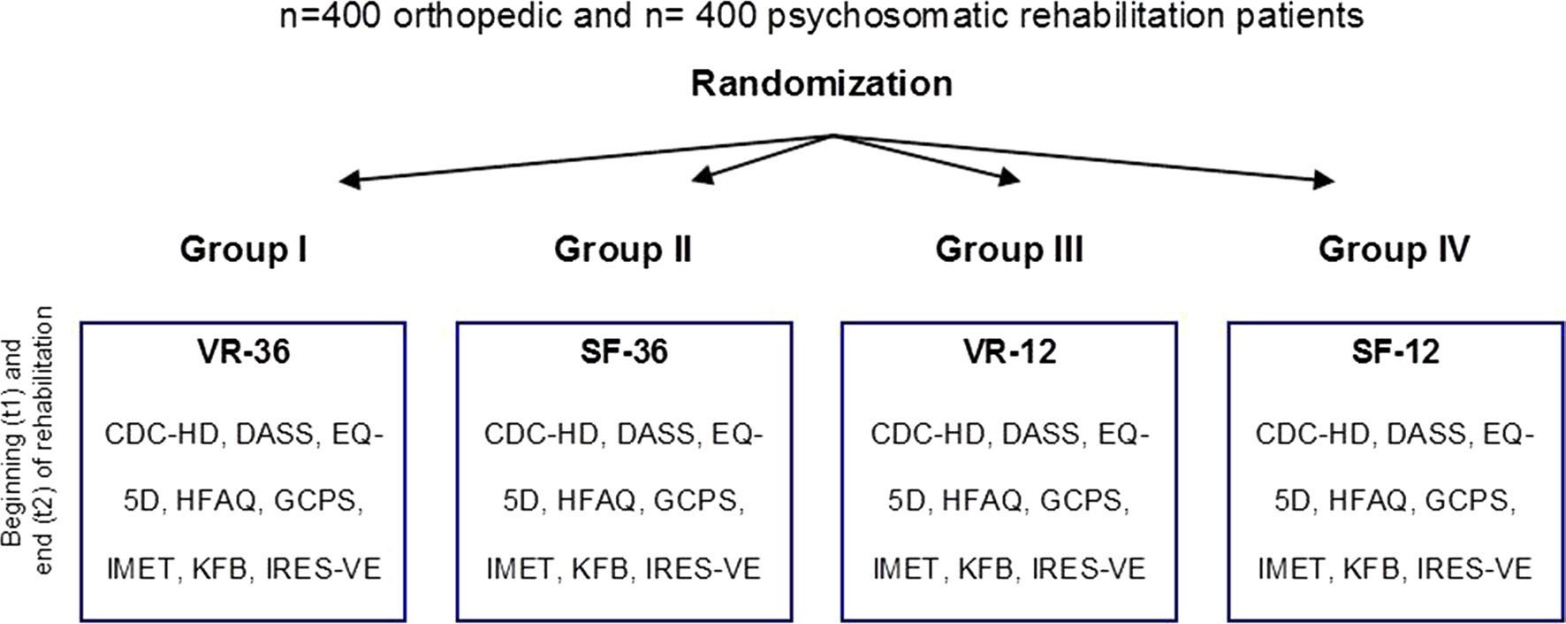
Survey study design

Study participants were rehabilitation patients undergoing a three- or six-week inpatient rehabilitation due to an orthopedic or a psychosomatic indication. Recruitment took place in five rehabilitation clinics between October 2015 and November 2017. Patients who did not had cognitive or linguistic impairments were consecutively included in the study if they provided written informed consent. Participants completed questionnaires at the beginning (t1, baseline) and at the end (t2, three- to six-week follow-up) of their course of rehabilitation. Based on sample size calculations, which included drop-out-assumptions of 20%, a study sample of n = 800 patients at t1 (n = 400/clinical indication and n = 200/instrument version) and n = 640 patients at t2 (n = 320/clinical indication and n = 160/instrument version) was targeted. Because the SF-36, the VR-36, the SF-12 and the VR-12 questionnaires are very similar, participants were randomly assigned to one of four groups (block-randomization) to complete only one of these instruments (Fig. 2). By block-randomization an indirect comparison between the long- and the short-forms of the VR and the SF could be made.

The study was approved by the ethics committee of the University Medicine Greifswald, Germany, and was conducted according to the Declaration of Helsinki.

## Measures

In addition to the VR and SF instruments, the patient questionnaires contained several other self-report measures. These measures were chosen to correspond to the eight scales and the summary scores of the VR instruments in order to validate the VR instruments.

The EQ-5D-5L questionnaire is an internationally widely used preference-based measure of self-assessed health [44–46]. The questionnaire measures impairments in five dimensions of health using five items, each with five levels of impairments, and a thermometer-like visual analogue scale (EQ VAS). The values of the five items can be converted into a preference-based single utility index. In the present study, index values were calculated using the German tariff [47].

The Centers for Disease Control and Prevention (CDC) “Healthy Days” is a generic HRQoL questionnaire containing four items measuring self-rated health and the number of disability days (out of the last 30) due to physical and mental health or limitations in activities [48, 49]. The instrument is valid and reliable [48].

The Hannover Functional Abilities Questionnaire (HFAQ) is a 12-item generic measure of (physical) functional ability of daily activities [50–52]. Each item has three levels of functioning. All items can be combined to an additive summary score.

The Depression, Anxiety and Stress Scale (DASS) is an extensively validated measure of mental health [53, 54]. In this study, the short form (21-item, DASS-21) instrument was used.

The Graded Chronic Pain Scale (GCPS) is an internationally established instrument developed by van Korff et al. [55, 56]. The GCPS measures self-rated pain intensity and pain disability using a 0 to 10 numeric rating scale plus one item regarding number of disability days (in the past three months) due to pain using seven items. Summation of GCPS items produce scores describing pain intensity and pain disability.

The Index for the Assessment of Health Impairments, IMET [57, 58], measures participation as defined by the WHO International Classification of Functioning, Disability and Health, ICF. The 9-item questionnaire was applied and tested in several samples from rehabilitation patients of different clinical indications. It is suitable as a screening method to assess the risk of a failure in the professional reintegration of rehabilitation patients. The instrument is demonstrated to be an economic, highly practicable, valid and reliable operationalization of “activities and participation” according to the concept of the ICF. Norm values for the IMET were assessed in a random sample of Lübeck inhabitants comprising subjects between 19 and 79 years of age, and enable classification of limitations in participation for people undergoing rehabilitation or suffering from chronic diseases.

The vitality subscale of the Indicators of the REhabilitation Status (IRES-VE) was included to examine the construct validity of the VR items on vitality [59]. In Germany, the IRES is recommended (in addition to the SF-36) for rehabilitation research and practice [42].

### Statistical analysis

The VR-36 and the VR-12 were analyzed regarding the completeness of data on the scale-level, distributional properties, construct validity, known-groups validity, internal consistency (as one aspect of reliability), and responsiveness to change. This was done on the summary scores of the VR-36 and the VR-12 (physical component score (PCS_VR_) and mental component score (MCS_VR_)) as well as the eight VR-36 scales: (physical functioning (PF_VR-36_), role functioning/physical (RP_VR-36_), role functioning/emotional (RE_VR-36_), vitality (VT_VR-36_), mental health (MH_VR-36_), social functioning (SF_VR-36_), pain (BP_VR-36_), and general health (GH_VR-36_)). The VR instruments have not previously been used in German populations and normed scores have not yet been developed. Therefore, summary scores and scales were scored according to the VR-36 and VR-12 algorithms, using a t-score transformation with a mean of 50 and a standard deviation of 10 and normed to a general sample of the US population for the summary scales (PCS and MCS) [23, 60–62]. The scoring algorithms for the VR-36 and the VR-12 impute for missing data. VR-12 extrapolates scoring based on the missing pattern; VR-36 conducts mean imputation at the subscale level if less than 50% of the subscale items is missing. In all analyses, all available data were used (available case analysis). Because the SF-36 and the SF-12 instruments are well validated across a range of populations, they were used as the comparator to the VR instruments for all analyses.

*Completeness of data* is an indicator of data quality and acceptance of the questionnaire by respondents. The percentage of non-missing responses was calculated for the eight VR-36 scales, stratified by respondent characteristics (e.g. clinical indication, age, sex, education). No imputation was carried out to deal with missing data for statistical analyses.

*Distributional properties* (such as means, standard deviations and range) for the VR instruments were analyzed on the scale and summary score levels. To compare the distributional properties of the PCS and MCS for both the VR-12 and SF-12 as well as the VR-36 and the SF-36, classical statistical indices of distribution such as mean, standard deviation, minimum, maximum, skewness (to assess and compare the type and strength of symmetry) and kurtosis (as a measure of the steepness / flatness of the frequency distribution) were assessed. Kolmogorow-Smirnov-test was used to compare the distributions of the two summary scores of the VR and the SF—i.e. PCS_VR_ and PCS_SF_ as well as MCS_VR_ and MCS_SF_. Kernel density plots using the Epanechnikov function were used to visually examine distribution of summary scores and scales.

*Construct validity* refers to the degree of accuracy with which a measurement instrument captures the construct it claims to measure. To examine construct validity, Pearson correlation coefficients (r_p_) between VR summary scores (PCS_VR_ and MCS_VR_) and other self-completed health measures were assessed. We compared these to the correlations between the PCS_SF_ and MCS_SF_ with other self-completed health measures. Correlation coefficients were compared using significance tests for correlations for independent samples [63]. The correlations between PCS_VR_ and other self-reported physical health measures (e.g. HFAQ, CDC Physical unhealthy days, GCPS Disability) were expected to be higher (convergent validity) than with self-report measures of mental health (divergent validity). Similarly, MCS_VR_ is expected to be more strongly correlated with self-reported mental health measures (e.g. DASS-Anxiety, DASS-Stress, DASS-Depression, CDC Mental unhealthy days) than with physical measures. Both PCS and MCS are expected to be similarly correlated with generic self-report measures (e.g. EQ VAS, IMET) and GCPS-Pain. Correlations were interpreted as follows: r_p_ < 0.1 small, 0.3 ≥ r_p_ < 0.5 moderate, r_p_ ≥ 0.5 high/strong [64].

*Known-groups validity* is a criteria-based technique to investigate the ability of a measure to discriminate between groups known to differ in the construct of interest. For this study, known-groups were defined by clinical indication (psychosomatic, orthopedic), treatment program (“curative therapy” typically for chronically ill patients, “medical follow-up treatment” generally after joint replacement, only for orthopedic patients) age (< 45 years, 45–65 years, > 65 years), duration of rehabilitation (median), sick days in the past 12 month, self-rated health (SRH, “excellent/very good/good” vs. “fair/poor”). We examined if mean PCS_VR_ and mean MCS_VR_ scores were significantly different between those pre-defined groups using t-tests for two groups or ANOVA for more than two groups.

*Internal consistency (IC)* is a measure of reliability. A scale is considered reliable if its items are homogeneous—i.e., highly correlated because they measure the same underlying construct [65]. In this study, Cronbach's alpha was used as a measure of IC with α ≥ 0.7 interpreted as acceptable, α ≥ 0.8 as good, and α ≥ 0.9 as excellent.

*Responsiveness* refers to a self-assessed health instrument’s ability to capture changes in health over time [66]. The raw difference of SF and VR summary scores from t1 to t2 were divided by the pooled standard deviation of change to produce standardized response means (SRM), or divided by baseline standard deviation to produce standardized effect size (SES). As we assess patients before and after an intensive treatment, analysis were restricted to respondents who reported stable (t1 = t2) or improved (t1 < t2) health on a single SRH item (n = 133) to assess responsiveness to health improvements. We further checked improvement (from t1 to t2) for all PCS- and MCS-scores of all four instruments using paired t-tests. The magnitude of changes in scores (expressed as SRM and SES) was interpreted as following: values of < 0.3 were considered as small, values between 0.3 and 0.59 were considered as medium, and values ≥ 0.6 were considered as large [67]. Since there are different methods to estimate the magnitude of change within groups, and consensus is lacking on their interpretation [68], we are calculating both SES and SRM for comparison purposes. Due to the repeated measurement design the measurements are correlated, which was shown to affect the magnitude of SRM [69]; to account for this, we additionally correlated both measurements (Pearson correlation coefficient, r_t1/t2_).

Data were analyzed using IBM SPSS Statistics 24 and STATA SE 13. Wherever applicable, analyses were stratified by clinical indication (orthopedic or psychosomatic rehabilitation).

## Results

### (A) Translation and cultural adaptation of the German VR

There were no major problems found in the forward–backward-translations. Reconciliation of the items did not lead to problems. The field test yielded that most of the questions (except for RE and RP instructions, response scales and questions) of the VR-36 are clear and simple to both rehabilitation patients (n = 15, 4 male, 11 female, 30 to 80 years (mean 55.3 years)) and patients from general practice (n = 11, 25 to 77 years (mean 57.4 years)) of all ages. Additional file 1 shows the key issues that were discussed during the translation process (forward–backward translation, reconciliation and cognitive debriefing) and how the items were reconciled. Besides the already described adaptation needs identified during the cognitive debriefings, adaptations to the cultural context were needed. The German SF-36 was used as a guide in these decisions. For example, playing golf (used as example in one item) is a less popular activity in Germany than for the USA. In the considerations for a culturally appropriate counterpart, hiking and walking were found to be appropriate but not practicable. We therefore removed the example as was also done for the German SF-36. In two items (BP2, SF1), for purposes of international equivalency, the right-most response category “extremely” was translated into German as “sehr” (English: “very much”), which is also used by the German version of the SF-36.

During the translation process, some double negatives were introduced as a result of combining the questions with their response choices (e.g. “[…] nicht so lange […]” (part of the question) “nein, nie” (response option)). As these double negatives also exist in the English version of the instruments, they were left in the German translation. However, nearly every third field-test participant had problems with the double negatives. Therefore, “yes” and “no” were omitted for these response categories to clarify the language. From a linguistic point of view, these revised response categories resemble the English SF-36 Version 2 and the German SF-36 (versions 1 and 2).

The final German VR-36 is conceptually identical to the English original.

### Phase (B) validation study

At t1, data are available from n_t1_ = 399 orthopedic (response: 99.8%) and n_t1_ = 292 psychosomatic (73%) rehabilitation patients. From n_t2_ = 378 of the 399 orthopedic (94.7%) and n_t2_ = 248 of the 292 psychosomatic (84.9%) patients data are also available for follow-up. Due to block-randomization, number and sample characteristics of participants were balanced across all four groups (n_VR-36_ = 169, n_SF-36_ = 174, n_VR-12_ = 177, n_SF-12_ = 171, Table 1). Study participants were on average 53 ± 10.6 (20–89) years old; 67.7% were women and 48.3% were fully employed. About every fourth participant (26.8%) completed high school. Average duration of inpatient rehabilitation (for their primary diagnosis) was 22 days for orthopedic and 35 days for psychosomatic patients (overall mean = 27.5 days). There were no systematic differences in the self-reported health status at baseline between the four study arms (CDC general health status p(χ^2^) > 0.05). Socio-demographic and clinical characteristics were comparable across the four arms of the study, which allowed for indirect comparisons (Table 1). The most common primary diagnosis were diseases of the musculoskeletal system and connective tissue (ICD-10: M00-M99: 48.9%), affective disorders (ICD-10: F30.0-F39-0: 19.8%) and neurotic, stress and somatoform disorders (ICD-10: F40.0-F49.0: 13.6%).

**Table 1:** Sample characterization at baseline (t1)

	VR-12	SF-12	VR-36	SF-36	Total
*Sample size, n*_*t1*_*/n*_*t2*_					
Total	177/151	171/156	169/158	174/161	691/626
Orthopedics	103/96	99/91	97/95	100/96	399/378
Psychosomatics	74/55	72/65	72/63	74/65	292/248
Age, M ± SD (range)	52.0 ± 11.3 (23–89)	53.2 ± 11.3 (22–84)	54.1 ± 8.9 (23–78)	52.0 ± 10.7 (20–77)	52.8 ± 10.6 (20–89)
Sex, % women	68.0	69.6	68.1	65.1	67.7
*Marital status, %*					
Single	13.4	13.9	8.6	17.3	13.3
Married/living with partner	65.1	67.5	65.6	63.7	65.5
*Highest school graduation, %*					
High school	28.8	25.2	24.3	28.7	26.8
Secondary school (10 years)	56.5	55.6	53.8	53.4	54.8
*Employment status, %*					
Fully employed	45.8	48.0	49.1	50.6	48.3
Pension application, % yes	10.2	11.3	8.5	14.9	11.3
Duration of rehabilitation, mean days	26.8	28.2	27.4	27.7	27.5
Sick leave, days in the last year, M ± SD	130 ± 162	121 ± 162	118 ± 152	109 ± 2138	119 ± 153

*M* mean, *SD* standard deviation, *SF-12* Short Form 12 Items Health Survey, *SF-36* Short Form 36 Items Health Survey. All group comparisons were not significant (p > 0.05)

### Completeness of data

Missing values were acceptable (< 7%) for the VR-36 and comparable to missing data patterns of the SF-36 (Table 2). The scale GH had the lowest percentage of completion for both the SF-36 (93.1%) and VR-36 (93.5%). As expected, there is a tendency of missing values to increase with increasing age and lower education.

**Table 2:** Percent complete items in each scale by instrument and patient subgroup

	Instrument (n)	PF	RP	BP	GH	VT	SF	RE	MH
*Total*	VR-36 (n = 169)	94.7	93.5	97	93.5	96.4	98.2	95.3	95.9
	SF-36 (n = 174)	95.4	97.7	96.6	93.1	96.6	97.7	98.9	96.6
*Clinical indication*									
Orthopedic	VR-36 (n = 97)	93.8	91.8	96.9	92.8	95.9	97.9	93.8	92.8
	SF-36 (n = 100)	94	98	97	90	95	97	99	95
Psychosomatic	VR-36 (n = 72)	95.8	95.8	97.2	94.4	97.2	98.6	97.2	100
	SF-36 (n = 74)	97.3	97.3	95.9	93.2	98.6	98.6	98.6	98.6
*Sex*									
Female	VR-36 (n = 111)	91.9	94.6	96.4	94.6	96.4	98.2	94.6	96.4
	SF-36 (n = 112)	94.6	97.3	95.5	87.5	95.5	97.3	98.2	95.5
Male	VR-36 (n = 52)	100	90.4	98.1	92.3	96.2	98.1	96.2	94.2
	SF-36 (n = 60)	96.7	97.3	98.2	98.3	98.3	98.3	100	98.3
*Age*									
≤ 45 years	VR-36 (n = 26)	96.2	96.2	100	92.3	100	100	96.2	100
	SF-36 (n = 41)	97.6	95.1	95.1	92.7	97.6	97.6	97.6	97.6
46–64 years	VR-36 (n = 114)	94.4	92.7	96.8	95.2	96	97.6	96	95.2
	SF-36 (n = 115)	96.5	99.1	97.4	93.4	97.4	98.3	99.1	97.4
≥ 65 years	VR-36 (n = 19)	94.7	94.7	94.7	84.2	94.7	100	89.5	94.7
	SF-36 (n = 18)	88.9	94.4	94.4	88.9	88.9	94.4	100	88.9
*Education*									
≤ 10 years	VR-36 (n = 115)	93	91.3	95.7	92.2	95.7	98.3	93	93.9
	SF-36 (n = 117)	94.9	98.3	95.7	91.5	95.7	97.4	99.1	95.7
> 10 years	VR-36 (n = 46)	97.8	97.8	100	95.7	97.8	97.8	100	100
	SF-36 (n = 55)	96.4	96.4	98.2	94.5	98.2	98.2	98.2	98.2

*PF* Physical functioning, *RP* Role physical, *BP* Bodily pain, *GH* General health, *VT* Vitality, *SF* Social functioning, *RE* Role emotion, *MH* Mental health, *n* sample size

### Distributional properties

Table 3 gives the distributional properties of the PCS and MCS for the VR and SF short and long form versions. Means, standard deviations and ranges for PCS were very similar between SF-36 and VR-36, SF-12 and VR-12. For MCS, mean differences (e.g. mean VR-36: 36.2, mean SF-36: 39.0), skewness, kurtosis, minimum and maximum of the distribution were larger between the SF-36 and VR-36 than between the SF-12 and VR-12.

**Table 3:** Distribution properties of PCS and MCS by instrument and version

Instrument	n	M ± SD	Min–Max	Kurtosis	Excess	K–S-Test
PCS_VR-36_	155	38.50 ± 10.15	11.7–67.8	0.151	− 0.226	**0.200**
MCS_VR-36_	155	36.18 ± 14.21	12.9–66.6	0.437	− 0.817	0.049
PCS_VR-12_	173	36.30 ± 11.55	12.7–59.3	0.141	− 0.969	**0.057**
MCS_VR-12_	173	37.23 ± 13.82	8.4–70.2	0.389	− 0.527	0.001
PCS_SF-12_	150	36.95 ± 10.95	15.5–61.6	0.27	− 0.724	**0.060**
MCS_SF-12_	150	39.04 ± 12.33	17.6–65.4	0.268	− 1.001	0.002
PCS_SF-36_	168	37.50 ± 11.67	13.8–66.1	0.289	− 0.465	**0.097**
MCS_SF-36_	168	39.03 ± 15.62	2.04–73.2	0.055	− 0.989	0.005

*K-S-Test* Kolmogorov-Smirnov-Test, *M* mean, *SD* standard deviation, *n* sample size

For the long and the short form versions of the VR and the SF, the PCS has normal distributions (p = 0.057 to 0.097) while the MCS does not (p < 0.05, Table 3). The findings do not substantively change when stratified by study arm and clinical indication (results not shown).

**Fig. 3:**
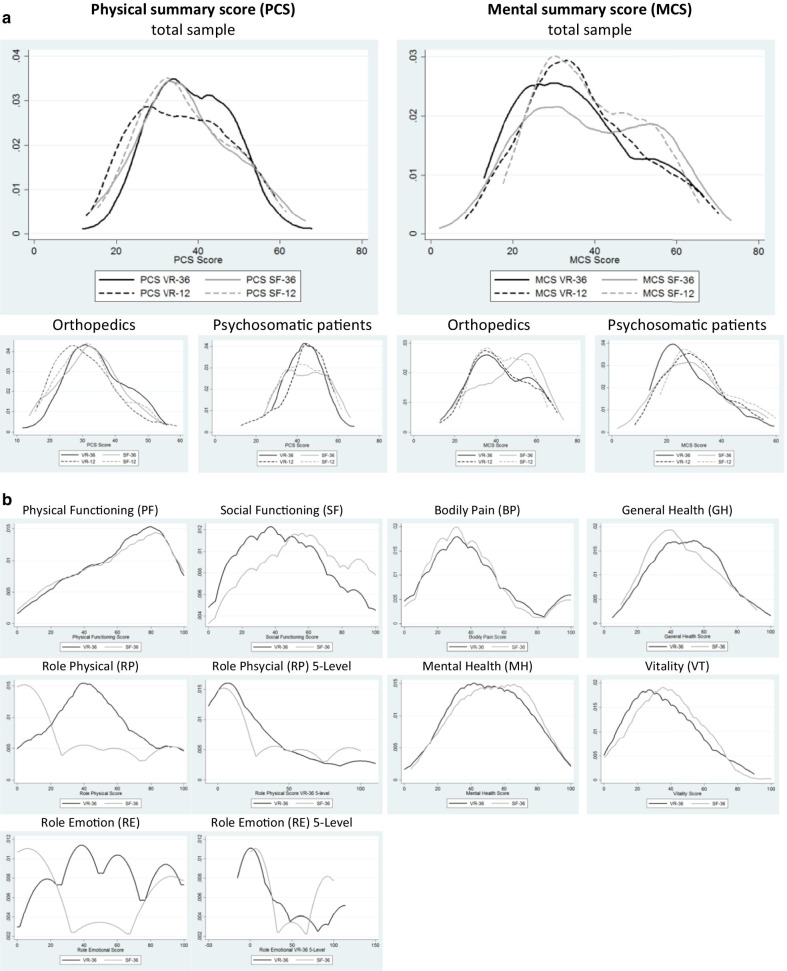
**a** Kernel density estimation for PCS and MCS **b** Kernel plots of the scales of the VR-36 and SF-36

The VR-36 scales distribute toward slightly lower scores than the SF-36 on the MCS, but not for the PCS. Kernel density plots show that the four instruments were more similar in PCS for orthopedic and MCS for psychosomatic patients. The distributions were more similar between the SF-12 and the SF-36 than between the SF and the VR instruments in PCS for psychosomatic and MCS for orthopedic patients (Fig. 3a). Differences were observed after stratifying by clinical indication. For the scales of the instruments, kernel plots of the VR-36 and the SF-36 are comparable for PF and BP, RP and RE, while kernel plots of SF_VR-36_, VT_VR-36_ and MH_VR-36_ are slightly more left-skewed compared to the SF-36 (Fig. 3b).

### Construct validity

Table 4 presents the correlations between VR and SF component scores and other self-reported measures. Moderate to strong correlations were observed between convergent measures with similar correlations observed across the VR-12 and SF-12, and VR-36 and SF-36. Differences (∆) of correlations between corresponding measures (PCS: HFAQ, CDC healthy days physical unhealthy days; MCS: DASS, IRES-VT, CDC mental unhealthy days) were below r_p_ = 0.090 and with one exception (IRES-VT vs. MCS for the short versions) statistically not significant (p > 0.5).

**Table 4:** Construct validity: comparison of Pearson correlation coefficients (r_p_) across SF-12/VR-12 and SF-36/VR-36

Physical component score (PCS)	PCS_SF-12_	PCS_VR-12_	∆_SF-12-VR-12_	p-value	PCS_SF-36_	PCS_VR-36_	∆_SF-36-VR-36_	p-value
*Generic*								
EQ VAS	0.480	0.327	0.153	0.052	0.348	0.328	0.020	0.420
IMET	− 0.499	− 0.514	0.015	0.429	− 0.362	− 0.457	0.095	0.155
GCPS Pain	− 0.514	− 0.620	0.106	0.082	− 0.420	− 0.412	− 0.008	0.466
*Convergent*								
HFAQ	0.670	0.759	− 0.089	0.052	0.746	0.660	0.086	0.064
CDC Physical healthy days	− 0.604	− 0.669	0.065	0.165	− 0.569	− 0.554	− 0.015	0.423
GCPS Pain Intensity	− 0.547	− 0.624	0.077	0.149	− 0.664	− 0.632	− 0.032	0.312
GCPS Pain Disability	− 0.581	− 0.615	0.034	0.319	− 0.661	− 0.586	− 0.075	0.137
*Divergent*								
DASS Anxiety	− 0.156	0.003	− 0.159	0.085	0.143	− 0.094	0.237	0.017
DASS Depression	0.023	0.124	− 0.101	0.183	0.164	− 0.005	0.169	0.065
DASS Stress	0.063	0.109	− 0.046	0.340	0.297	0.104	0.193	0.036
IRES-VT	0.106	− 0.082	0.188	0.415	− 0.151	− 0.015	− 0.136	0.111
CDC Mentally healthy days	0.174	0.119	0.055	0.309	0.234	0.118	0.116	0.493

Mental component score (MCS)	MCS_SF-12_	MCS_VR-12_	∆_SF-12-VR-12_	p-value	MCS_SF-36_	MCS_VR-36_	∆_SF-36-VR-36_	p-value
*Generic*								
EQ VAS	0.311	0.321	− 0.010	0.461	0.282	0.451	− 0.169	0.041
IMET	− 0.420	− 0.426	0.006	0.474	− 0.392	− 0.490	0.098	0.139
GCPS Pain	− 0.172	− 0.062	− 0.110	0.162	0.013	− 0.080	0.093	0.204
*Divergent*								
HFAQ	0.054	− 0.137	0.191	0.045	− 0.141	0.036	− 0.177	0.057
CDC Physical healthy days	− 0.046	0.064	− 0.110	0.166	0.020	− 0.146	0.166	0.069
GCPS Pain Intensity	− 0.154	− 0.013	− 0.141	0.069	0.091	− 0.100	0.191	0.044
GCPS Pain Disability	0.072	− 0.031	0.103	0.182	0.022	− 0.159	0.181	0.052
*Convergent*								
DASS Depression	− 0.772	− 0.729	− 0.043	0.192	− 0.788	− 0.734	− 0.054	0.126
DASS Anxiety	− 0.524	− 0.596	0.072	0.177	− 0.603	− 0.609	0.006	0.466
DASS Stress	− 0.673	− 0.702	0.029	0.314	− 0.796	− 0.729	− 0.067	0.076
IRES-VT	0.701	0.784	− 0.083	0.050	0.806	0.763	0.043	0.159
CDC Mentally healthy days	− 0.744	− 0.746	0.002	0.484	− 0.684	− 0.704	0.020	0.366

*CDC* healthy days, *EQ-5D-5L* EuroQol-5 Dimensions, *HFAQ* Hannover Functional Ability Questionnaire, *IMET* Index of the Assessment of Health Impairments, *IRES-VT* Subscale Vitality of the Indicators of Rehabilitation Status, *GCPS* Graded Chronic Pain Scale, *SF-12* Short-Form 12 Items Health Survey, *SF-36* Short Form 36 Items Health Survey, *VR-12* Veterans Rand 12 Items Health Survey, *VR-36* Veterans Rand 36 Items Health Survey; p-value for the comparison of two correlation coefficients from independent samples [63]

The PCS_VR_ had moderate to strong correlations (r_p_ = 0.33 to r_p_ = 0.62) with generic measures and strong correlations (r_p_ = -0.55 to r_p_ = 0.76) with physical health measures. The MCS_VR_ had moderate correlation with generic health measures (r_p_ = 0.32 to r_p_ = 0.49) and strong correlations with mental health measures (r_p_ = -0.60 to r_p_ = 0.78).

At r_p_ =  -0.5 (PCS_SF-12_) and r_p_ = -0.6 (PCS_VR-12_), the correlation between the short versions of the PCS and the GCPS Pain was greater than for the long versions (both PCS_SF-36_ and PCS_VR-36_ r_p_ = -0.4). The MCS of all versions was almost uncorrelated with the GCPS Pain (r_p_ = -0.172 to r_p_ = 0.013).

### Known-groups validity

Table 5 illustrates the PCS_VR-36_ and MCS_VR-36_ scores in sub-samples of known groups. Lower mean PCS_VR-36_ was found for orthopedic patients while lower mean MCS_VR-36_ was found for psychosomatic patients. In line with our hypothesis, higher mean PCS_VR-36_ and MCS_VR-36_ scores were found in younger patients with fewer sick days in the last year and a shorter duration of rehabilitation. As expected, at baseline, orthopedic patients reported better mental health compared to psychosomatic patients and the other way around for mental health, which is reflected by higher mean MCS_VR-36_ scores in orthopedic and higher mean PCS_VR-36_ scores in psychosomatic patients. Results were similar for VR-12 and VR-36 suggesting that both instruments perform similarly with respect to known-groups validity (Table 6): all MCS and PCS scales differentiated groups based on clinical indication, duration of rehabilitation and self-rated health, PCS_VR-12_ additionally for sick days. As this is only applicable for orthopedic patients, both PCS scales additionally differentiated for type of therapy.

**Table 5:** Known groups validity for the PCS_VR-36_ and the MCS_VR-36_

Subgroups	PCS_VR-36_		MCS_VR-36_	
	mean ± SD	p-value	mean ± SD	p-value
*Clinical Indication*		< 0.001		< 0.001
Orthopedic patients (n = 87)	34.2 ± 9.0		41.9 ± 13.9	
Psychosomatic patients (n = 68)	44.1 ± 8.8		28.9 ± 11.0	
*Type of therapy*^a^		< 0.001		0.149
Medical follow-up-treatment (n = 30)	29.6 ± 8.9		44.1 ± 13.9	
Curative treatment (n = 54)	36.7 ± 8.1		39.6 ± 13.3	
*Age*		0.181		0.133
< 45 years (n = 25)	41.3 ± 8.4		31.8 ± 13.6	
45–65 years (n = 114)	38.3 ± 10.7		37.5 ± 14.4	
> 65 years (n = 16)	35.5 ± 8.0		33.5 ± 12.9	
*Duration of rehabilitation*		< 0.001		< 0.001
≤ 27 days (n = 71)	35.2 ± 9.2		40.7 ± 13.5	
> 27 days (n = 66)	42.4 ± 10.3		32.5 ± 13.1	
*Sick days*^b^		0.081		0.281
≤ 100 days (n = 76)	40.3 ± 10.6		38.1 ± 14.1	
> 100 days (n = 66)	37.3 ± 9.7		35.6 ± 14.0	
*SRH*^c^		< 0.01		< 0.001
Excellent/very good/good (n = 60)	42.7 ± 11.0		43.4 ± 13.3	
Fair/poor (n = 92)	35.7 ± 8.7		31.8 ± 13.0	

^a^Orthopedic patients only

^b^Days of sick leave in the last 12 month

^c^SRH Self-rated health. Patients reporting “excellent”, “very good” or “good” health and those reporting “poor” or “fair” health were aggregated. SD standard deviation

**Table 6:** Known groups validity for the PCS_VR-12_ and MCS_VR-12_

Subgroups	PCS_VR-12_		MCS_VR-12_	
	mean ± SD	p-value	mean ± SD	p-value
*Clinical Indication*		< 0.001		< 0.001
Orthopedic patients (n = 100)	31.20 ± 9.49		42.21 ± 13.73	
Psychosomatic patients (n = 73)	43.13 ± 10.55		30.41 ± 10.74	
*Type of therapy*^a^		0.023		0.187
Medical follow-up treatment (n = 37)	28.37 ± 9.67		44.56 ± 15.34	
Curative therapy (n = 67)	32.88 ± 9.20		40.58 ± 12.42	
*Age*		0.079		0.004
< 45 years (n = 42)	37.15 ± 11.77		35.84 ± 14.14	
45–65 years (n = 111)	36.86 ± 11.37		36.02 ± 12.94	
> 65 years (n = 20)	30.78 ± 11.13		46.87 ± 14.77	
*Duration of rehabilitation*		< 0.001		< 0.001
≤ 27 days (n = 85)	31.69 ± 10.74		42.45 ± 13.64	
> 27 days (n = 71)	41.77 ± 9.91		31.89 ± 11.64	
*Sick days*^b^		0.021		0.667
≤ 100 days (n = 82)	38.56 ± 10.71		37.58 ± 13.66	
> 100 days (n = 82)	34.41 ± 12.04		36.65 ± 13.96	
*SRH*^c^		< 0.001		< 0.001
Excellent/very good/good (n = 67)	41.56 ± 11.49		43.42 ± 13.81	
Fair/poor (n = 104)	32.38 ± 10.19		33.50 ± 12.37	

^a^Orthopedic patients only

^b^Days of sick leave in the last 12 month

^c^SRH Self-rated health. Patients reporting “excellent”, “very good” or “good” health and those reporting “fair” or “poor” health were aggregated. SD standard deviation

### Internal consistency (IC)

Except for GH (acceptable), IC was good to excellent for both VR and SF scales and with one exception (MH) always higher for the VR scales (Table 7).

**Table 7:** Cronbachs α in each scale by instrument

Instrument (n^b^)	Scale (number of items)							
	PF (10)	RP (4)	BP (2)	GH^a^ (5)	VT (4)	SF (2)	RE (3)	MH (5)
VR-36 (n = 164)	0.92	0.93	0.89	0.79	0.85	0.87	0.94	0.87
SF-36 (n = 172)	0.92	0.85	0.88	0.70	0.82	0.80	0.89	0.90

^a^Without GH item 1 α_VR-36_ = 0.75, α_SF-36_ = 0.66

^b^Due to missing values, n varies between n = 158 and n = 164 (of n = 169) for the VR-36 and n = 159 to n = 172 (of n = 174) for the SF-36

### Responsiveness

Responsiveness to change analysis included the n = 50 to n = 88 cases with no deterioration in SRH from t1 to t2, stratified as necessary by study arm (Table 8). For PCS, SES varied from 0.102 (VR-36 psychosomatic) to 0.398 (SF-12 orthopedic) and SRM varied from 0.127 (VR-36 psychosomatic) to 0.695 (VR-12 orthopedic) with better responsiveness across all instruments for orthopedic patients. Effect sizes of the short versions (VR-12, SF-12) were larger than those of the long versions (VR-36, SF-36). In psychosomatic patients, responsiveness to change of MCS was at least twice as large as responsiveness of PCS, while in orthopedic patients there were less obvious differences in responsiveness to change between PCS and MCS. Responsiveness of the PCS_VR-36_ for psychosomatic patients was smaller than the other instruments. Score improvements for all four instruments were statistically significant at p < 0.001 (paired t-tests).

**Table 8:** Standardized response means (SRM) and standardized effect sizes (SES) by instrument and clinical indication

Version	Orthopedics						Psychosomatics					
		SES	SRM	r_t1/t2_		SES	SRM	r_t1/t2_				
	n_i/s_ (n_c_)	PCS	MCS	PCS	MCS	PCS/MCS	n_i/s_ (n_c_)	PCS	MCS	PCS	MCS	PCS/MCS
VR-36	78 (82)	0.235	0.298	0.328	0.452	0.74/0.77	56 (61)	0.102	0.522	0.127	0.527	0.65/0.62
SF-36	83 (88)	0.317	0.165	0.460	0.241	0.77/0.74	59 (60)	0.250	0.818	0.331	0.820	0.72/0.54
VR-12	88 (93)	0.381	0.357	0.545	0.586	0.73/0.81	50 (57)	0.218	1.027	0.317	0.876	0.73/0.35
SF-12	63 (69)	0.398	0.482	0.695	0.586	0.84/0.65	50 (54)	0.277	0.840	0.357	1.060	0.69/0.69

*SES* standardized effect size, *SRM* standardized response mean, *r* Pearson correlation coefficient, *n*_*i/s*_ number of patients reporting improved or stable health defined by GHP1 (SRH, item 1 of the SF-36), *n*_*c*_ n complete: number of all cases (improved, deteriorated, unchanged), *PCS* physical summary score, *MCS* mental summary score

## Discussion

This research project (1) translated and culturally adapted the English VR-36 to the German language (Germany) and (2) validated the adapted VR-36 and VR-12 in German orthopedic and psychosomatic inpatient rehabilitation patients. This article provides details of the translation and cultural adaptation process of the German VR and the main findings of the validation study.

The German translation of the VR was prepared according to "state of the art" criteria for cultural adaptation of self-assessed health questionnaires using forward and backward translations. The study produced a self-report questionnaire that is conceptually and semantically equivalent to the English language VR-36. The only difficulty during translation was the role physical (RP) and role emotional (RE) items which produced double negatives when the question stems and responses were taken together. This was resolved by a slight change in response category wording.

The German VR-36 is the third cultural adaptation and translation of the VR after the Spanish and the Chinese version. Three more language versions (Japanese, Russian, Polish) are being planned.^1^

The validation phase of this study found the VR instruments to be acceptable, valid and moderately to strongly responsive to improvements in health. We indirectly compared the German VR-36 and VR-12 to the well-established SF-36 and SF-12, and found the instruments to be comparable in their distribution properties, validity, and responsiveness. Data quality indicators, such as the extent of item non-response, show the VR to be acceptable instruments in a German rehabilitation population, and were similar compared to the SF instruments. PCS score distributions were similar for VR and SF instruments. However, the MCS_VR_ was distributed more in the lower range of the scale than the MCS_SF_. The VR scales and summary scores were moderately to strongly correlated with expected external measures such as self-reported pain, physical functioning, mental functioning and disability. Both the long and the short form of the VR could distinguish between patient type (orthopedic and psychosomatic), duration of rehabilitation and self-rated health while both PCS_VR-12_ and PCS_VR-36_ could also distinguish between type of therapy and PCS_VR-12_ whether the patient had over 100 sick days in the last year. The short version (VR-12) was similarly responsive as the VR-36 and SF-36. Thus, the VR was established as a valid and responsive measure of quality of life in orthopedic and psychosomatic samples of German inpatient rehabilitation patients.

The number of studies using one of the instruments of the VR family is increasing every year with well over 400 publications [70]. The developers of the VR family provided the original psychometric evidence for the VR-36 and VR-12 [13, 15, 16, 23].

Item level missing values were low and comparable to other studies suggesting high acceptability. While in this study 1.8% to 6.5% were missing per question for the baseline VR-36, Kronzer et al. [71] reported missing values in adult patients undergoing elective surgery on the baseline VR-12 from 1.5 to 3.7% per question and from 3.3 to 8.9% on the follow-up VR-12 (median 56 days).

Descriptive statistics indicated acceptable distributional characteristics. Summary scale means and SD of the PCS_VR-36_ are comparable with the results of the Veterans Health Study (VHS), in which the VR-36 was administered to nearly 2,500 veterans receiving ambulatory care (VHS PCS_VR-36_: 37.12 ± 11.85, this study: 38.50 ± 10.2), but MCS_VR-36_ is different (VHS: 47.81 ± 12.23, this study: 36.2 ± 14.2) [17]. The differences in MCS may be a function of the populations sampled; while the means were different the SD are quite similar.

The validity results are comparable with other studies investigating physically impaired patients: a study with patients undergoing knee arthroplasty [31] found a moderate correlation between the PCS_VR-12_ and a disease-specific measure (KOOS-pain score: 0.57). Since only few studies investigated the factor structure of the VR-36, e.g. [60], this needs further investigation.

Oak et al. [31] found the PCS_VR-12_ to capture statistically significant improvements in n = 45 pre- and postoperatively tracked patients who underwent knee arthroplasty. They found no statistical differences in internal or external responsiveness to change among the EQ-5D, VR-12 and PROMIS 10 physical instruments with SRMs of the PCS_VR-12_ of 0.681 and for the MCS_VR-12_ of 0.103 (SRM EQ-5D: 0.704, PROMIS 10 physical: 0.721, PROMIS 10 mental: 0.083). SRM of VR-12 scores at baseline and at the end of therapy (0.549) can be calculated from results of Levy et al.’s study of physical therapy received through tele-rehabilitation [73]. This is extremely similar to what we found for the VR-12 in orthopedic patients. Bedigrew et al.’s [74] study of an orthotic and rehabilitation program found statistically significant improvements only in the PCS but not in the MCS. For orthopedic patients, we found PCS to be less sensitive to changes in both SF and VR than the MCS, with the VR-12 similar or more sensitive to improvements than the SF instruments. However, the VR 36 was found to be slightly less sensitive to improvements than the SF-36 for psychosomatic patients.

Although the VR-36 and VR-12 are based on version 1 of the SF-36 and SF-12, the VR instruments use the five-level response format of the role functioning and role emotional scales whereas the SF version 1 instruments use the two-level format. The SF version 2 uses five-level response scales for those scales, but has slightly different wording and is in general a different instrument than version 1. This difference is likely the source of differences in distribution and responsiveness in our comparison of the VR to SF version 1 instruments. The floor was raised and ceiling lowered with the 5-point set of response choices for the role physical and role emotional scales compared with the dichotomized choices for the SF version 1 instruments [16]. Previous findings suggest that this could also be a possible explanation for the differences in responsiveness [16]. Gornet et al. [35] investigated the conversion of the SF-36 to PCS_VR-12_ and MCS_VR-12_ in 1968 patients who underwent lumbar (n = 1559) and cervical (n = 409) surgery between 1998 and 2013. They found the SF-36 and converted VR-12 mean scores, the mean (pre to post) change scores for PCS and MCS, and the minimum detectable change (MDC) to be extremely similar. However, as their study only collected SF-36 data, they could not compare how a 2-level and 5-level response category in the two scales might differ.

The primary limitation of this study is the indirect comparison of the instruments: the VR-36, VR-12, SF-36 and SF-12 were completed by different patients. The design choice was to minimize respondent burden and frustration as the four instruments are very similar. Although patients were randomized to the study arms, there could be underlying differences across the groups not captured by demographic or patient characteristics. Thus, it is possible that the detected distribution and responsiveness differences may in part be due to differences in the sample characteristics and perhaps unmeasured variables and not due to the instruments themselves.

Due to the magnitude of this time interval (of four to six weeks) and the intervention, it was not feasible to investigate test-retest reliability. Even after a week, which is the usual lag time between test-retests, we would expect patients to change as they are undergoing intense rehabilitation treatment. This is why we investigated internal consistency as a measure of internal reliability. However, test-retest reliability it is still to be investigated for the German version of VR.

Furthermore, the German VR was validated in an inpatient rehabilitation setting, and the results may not be generalizable to other populations nor to outpatient rehabilitation settings. Future research applying the German VR in other settings is necessary. The instruments were also administered only as a paper-and-pencil survey. As self-assessment questionnaires are increasingly being used in electronic formats, the comparison between the classical paper-pencil and other new computer platform applications should be studied.

Since this is the first study to this new German instrument, which aimed to adapt and test it in the German population, German norms have not yet been developed. This will be one of the next steps of instrument development. Therefore, for evaluation for this study, we relied on the US norms.

## Conclusions

The VR is a credible measure in the public domain that can be applied in the German rehabilitation context. The VR measure may be appropriate for use in clinical research and clinical practice, but further research is needed to evaluate its usefulness in other populations in German. Due to the high demand for the German VR during the study period, it can be assumed that in the foreseeable future more data from different clinical settings and administrative modes will be available. The scoring algorithms also have been developed by the project working group for common statistical programs (e.g. SPSS, Stata, R) and is, as well as the questionnaires, freely available for use to the research community.

## Supplementary Information

**Additional file 1:** Key differences between the original English and the German Translated VR-36. This file provides information on the key differences between the original English VR and its German translation. It shows an extract of the translation protocol and helps the reader to identify and retrace main semantical and conceptual differences between both versions due to cultural and linguistic adaptations during the translation process.

## Data Availability

The datasets collected and analyzed during the current study are not publicly available but are available from the corresponding author on reasonable request. The German Version of the VR-12 and VR-36 as well as the scoring algorithms developed in this project are available by request to Prof. Lewis E. Kazis. The VR-36 and VR-12 surveys in the English version are copyright by the trustees of Boston University. More information at: https://www.bu.edu/sph/about/departments/health-law-policy-and-management/research/vr-36-vr-12-and-vr-6d/.

## Abbreviations

EQ-5D-5L: EuroQol-5 Dimensions 5 Level
EQ VAS: Visual Analogue Scale
DASS: Depression, Anxiety and Stress Scale
HFAQ: Hannover Functional Abilities Questionnaire
GCPS: Graded Chronic Pain Scale
SF-36: Short Form 36 Items Questionnaire
PCS: Physical summary score of the SF-36
MCS: Mental summary score of the SF-36
CDC: Healthy Days Centers for Disease Control and Prevention “Healthy Days”
GHP: General health perception
PF: Physical functioning
BP: Bodily pain
RP: Role physical
SF: Social functioning
MH: Mental health
RE: Role emotion
VT: Vitality
IMET: Index for the Assessment of Health Impairments
IRES-VT: Indicators of Rehab Status, subscale vitality
SES: Standardized effect size
SRM: Standardized response mean
n: Number of cases
M: Mean
SD: Standard deviation

## Footnotes

1 To request free access to the instruments, information on terms of use, name and institution can be found at Boston University’s website [67]. The scoring algorithms for SAS, R, SPSS and Stata are available from Prof. L.E. Kazis.
