# Supplementary material for: Translation and adaptation of the German version of the Veterans Rand—36/12 Item Health Survey
Source: Health Qual Life Outcomes. 2021 May 4;19:137. doi: 10.1186/s12955-021-01722-y (PMC8097879; doi:10.1186/s12955-021-01722-y)
Supplement: Supplementary file 1 — Additional file 1: Key differences between the original English and the German Translated VR-36. This file provides information on the key differences between the original English VR and its German translation. It shows an extract of the translation protocol and helps the reader to identify and retrace main semantical and conceptual differences between both versions due to cultural and linguistic adaptations during the translation process. [file 12955_2021_1722_MOESM1_ESM.docx]

**Additional file 1** Key differences between the original English and the German Translated VR-36

| Item | Original English VR | Translated German VR | Comment |
| --- | --- | --- | --- |
| PF02 | Moderate activities, such as moving a table, pushing a vacuum cleaner, bowling, or playing golf? | Mittelschwere Tätigkeiten, z.B. einen Tisch verschieben, staubsaugen, kegeln | In the considerations for a culturally appropriate counterpart, hiking and walking were considered linguistically appropriate, but not found practicable. Since an imbalance between household and leisure activities is caused by the removal of the example, it was discussed whether an additional item in the field of recreational activities should be added. It was decided to remove the example as in the revised German Version of the SF-36. |
| PF07 | Walking more than a mile? | mehr als einen Kilometer zu Fuß gehen | In Germany, distances are usually given in kilometers rather than miles. Conceptually, a distance of “more than one kilometer” (German: “mehr als einen Kilometer”) is nearly the same as “more than a mile”. This is identical to the revised English and German Version of the SF-36. |
| PF08 | Walking several blocks? | mehrere hundert Meter zu Fuß gehen | Conceptually, a distance of “several hundred meters” (German: “mehrere hundert Meter”) is nearly the same as “several blocks”. This is identical to the revised English and German Version of the SF-36. |
| PF09 | Walking one block? | einhundert Meter zu Fuß gehen | As with the SFv2: Cultural adaptation. Crossroads and blocks are in Germany no traditional distance unit. Thus, we circumscribed the distance of “one block” by using “one-hundred meters” (German: “einhundert Meter”), which is regional more common and conceptually nearly the same. |
| Instruction RP | During the past 4 weeks, have you had any of the following problems with your work or other regular daily activities as a result of your physical health?  NO, NONE OF THE TIME - YES, A LITTLE OF THE TIME - YES, SOME OF THE TIME - YES, MOST OF THE TIME - YES, ALL OF THE TIME | Hatten Sie in den vergangenen 4 Wochen aufgrund Ihrer körperlichen Gesundheit die folgenden Probleme bei der Arbeit oder bei anderen Alltagstätigkeiten?  nie – selten – manchmal – meistens – immer | Almost ^1^/_3_ of the participants in the field test had difficulties with the “yes”/”no” (German: “ja”/”nein”) in front of the main body of the response categories. Due to the “yes”/”no” words preceding frequency descriptors in the German translation, double negatives were introduced when the items and the response categories are read together.  It was suggested to omit “yes” and “no” for these response categories. From a linguistic point of view, double negatives are a more serious problem than the omission of “yes”/”no”. As “yes”/”no” is also not part of the response categories of the English SF-36 Version 2 and the German SF-36 and SF-12 Version I and II, it can be assumed that this will produce no major differences. |
| Instruction RE | During the past 4 weeks, have you had any of the following problems with your work or other daily activities as a result of any emotional problems (such as feeling depressed or anxious)?  no, none of the time – yes, a little of the time – yes, some of the time – yes, most of the time – yes, all of the time | Hatten Sie in den vergangenen 4 Wochen aufgrund seelischer Probleme die folgenden Schwierigkeiten bei der Arbeit oder bei anderen Alltagstätigkeiten (z.B. weil Sie sich niedergeschlagen oder ängstlich fühlten)?  nie – selten – manchmal – meistens – immer | See above. |
| Instruction SF1 | During the past 4 weeks, to what extent has your physical health or emotional problems interfered with your normal social activities with family, friends, neighbors, or groups?  NOT AT ALL – SLIGHTLY – MODERATELY - QUITE A BIT – EXTREMELY | Wie sehr haben Ihre körperliche Gesundheit oder seelischen Probleme in den vergangenen 4 Wochen Ihre normalen Unternehmungen mit der Familie, mit Freunden, Nachbarn oder dem Bekanntenkreis beeinträchtigt?  überhaupt nicht- etwas - mäßig – ziemlich – sehr | Result of the conference call with Boston University School of Public Health, Department of Health Law, Policy & Management: For purposes of international equivalency, the right-most response category “extremely” will be translated into German as “sehr” (English: “very much”), which is also used by the German version of the SF-36. |
| Instruction BP2 | During the past 4 weeks, how much did pain interfere with your normal work (including both work outside the home and house work)?  NOT AT ALL - A LITTLE BIT – MODERATELY - QUITE A BIT – EXTREMELY | Inwieweit haben Schmerzen in den vergangenen 4 Wochen Ihre normale Arbeit (im Beruf und zu Hause) beeinträchtigt?  überhaupt nicht – ein wenig – mäßig – ziemlich - sehr | Result of the conference call with Boston University School of Public Health, Department of Health Law, Policy & Management: For purposes of international equivalency, the right-most response category “extremely” will be translated into German as “sehr” (English: “very much”), which is also used by the German version of the SF-36. |
